# Gestational hypercholanemia suppresses pregnancy‐associated adipose mass increase and stimulates a pro‐inflammatory environment in mice

**DOI:** 10.14814/phy2.70141

**Published:** 2024-12-12

**Authors:** Vanya Nikolova, Alice L. Mitchell, Elena Bellafante, Eugene Jansen, Georgia Papacleovoulou, Per‐Olof Bergh, Hanns‐Ulrich Marshall, Catherine Williamson

**Affiliations:** ^1^ Department of Women and Children's Health, Guy's Campus King's College London London UK; ^2^ Department of Metabolism, Digestion and Reproduction, Hammersmith Campus Imperial College London London UK; ^3^ Centre for Health Protection, National Institute for Public Health and the Environment Bilthoven The Netherlands; ^4^ The Wallenberg Laboratory, Department of Molecular and Clinical Medicine, Sahlgrenska Academy University of Gothenburg Gothenburg Sweden

**Keywords:** adipocyte, fetal growth restriction, hypercholanemia, hyperlipidemia, intrahepatic cholestasis of pregnancy

## Abstract

Women with intrahepatic cholestasis of pregnancy (ICP) have hypercholanemia alongside an increased risk of dyslipidemia. We investigated how cholic acid (CA) supplementation in murine pregnancy impacts adipose tissue function. Mice were fed normal or 0.5% CA‐supplemented chow from identification of copulatory plug until gestational day 14 or 15 (*n* = 10–11/group) and were matched experimentally with nonpregnant mice (*n* = 7/group). Tissue weights were measured alongside plasma bile acids, glucose, lipids, reactive oxygen metabolites (ROM), and adipokines. Subcutaneous and gonadal adipocyte mRNA expression was evaluated. CA supplementation inhibited pregnancy‐associated adipose tissue expansion and decreased fetal weight. CA diet in pregnancy increased LDL‐cholesterol and reduced HDL‐cholesterol. Pregnancy and CA diet reduced lipid metabolism transcript expression in adipocytes. CA supplementation during pregnancy increased plasma ROM by 1.24‐fold and suppressed inflammatory‐modulating pentraxin‐2/3 and insulin‐like growth factor 1 (IGF‐1) levels by >50% and >80%, respectively. Together, we show that hypercholanemia disturbs pregnancy‐associated adipose tissue expansion and mRNA expression in late gestation concomitant with reduced IGF‐1, altered lipid availability and increased inflammation and oxidation, which could impact fetal growth. This work highlights the need to better understand adipose tissue and redox stress changes in ICP pregnancies and the potential implications for fetal health.

## 1 INTRODUCTION

Intrahepatic cholestasis of pregnancy (ICP) is a pregnancy‐specific liver disorder that typically occurs in the late second or third trimester of pregnancy. ICP is characterized by pruritus associated with raised total serum bile acids (hypercholanemia) often combined with elevated levels of circulating liver transaminases (Williamson & Geenes, 2014). ICP affects approximately 0.3%–15% of pregnancies worldwide, with prevalence varying according to both country and ethnic group (Floreani & Gervasi, 2016; Lee et al., 2006; Pusl & Beuers, 2007; Reyes, 1982). It is associated with an increased risk of adverse pregnancy outcomes, including spontaneous preterm labor, prolonged admission to the neonatal unit, meconium staining of the amniotic fluid and stillbirth (Geenes et al., 2014; Glantz et al., 2004; Ovadia et al., 2019).

Early pregnancy is considered anabolic as maternal adipose tissues increase fat storage, peaking near the end of the second trimester (Herrera, 2000). This occurs through adipocyte hyperplasia alongside increased lipogenesis. In the latter stages of pregnancy, adipose tissue switches to a catabolic state to increase lipolysis, reduce tissue glucose uptake and provide increased levels of circulating lipids and glucose for fetal growth (Trivett et al., 2021). As a consequence of increased lipogenesis and reduced lipolysis a mild hyperlipidemia is observed in uncomplicated human pregnancies; total, LDL‐ and HDL‐cholesterol and triglycerides are elevated as gestation progresses (Mulder et al., 2024). Women with ICP have further elevated serum lipids during pregnancy (Dann et al., 2006; Martineau et al., 2015) and have a 3‐fold increased risk of developing gestational diabetes mellitus (Martineau et al., 2014; Wikström Shemer et al., 2013), but are not typically overweight/obese (Ovadia et al., 2019). In murine pregnancy, while there is adipose tissue expansion (Zhang et al., 2011) and increased serum triglycerides, total cholesterol levels decrease as gestation advances (Nikolova et al., 2017).

Deficiency of the bile salt export pump (BSEP), encoded by *Abcb11*, leads to hypercholanemia. Prior to onset of hypercholanemia, *Abcb11* knockout mice show increased hepatic triglycerides and reduced mitochondrial fatty acid β‐oxidation. Furthermore, reduced adipose depot size was evident in *Abcb11* ^*−/−*^ mice alongside reduction in lipoprotein lipase mRNA expression, suggesting altered adipose tissue function in response to dysregulated bile acid homeostasis (Zhang et al., 2012). The double knockout mouse model for farnesoid X receptor (*Fxr*) and small heterodimer protein (*Shp*), proteins involved in regulating bile acid synthesis (Chiang, 2009), presents with bile acid overload. *Fxr* ^*−/‐*^ *Shp* ^*−/−*^ mice display reduced white and brown fat mass and adipocyte size (Zhou et al., 2023). Thus, disrupting bile acid homeostasis impacts adipose tissue size and function in nonpregnant mouse models. We hypothesized that bile acid overload in pregnancy would contribute to the dyslipidemic phenotype observed in hypercholanemic pregnancy due to interference in pregnancy‐related changes in adipose tissue function and signaling.

## 2 MATERIALS AND METHODS

### 2.1 Animal experiments

8‐week‐old C57BL/6J mice were purchased from Harlan Laboratories (UK). Mice were housed in a temperature and light‐controlled environment with 12‐h light/dark cycles with free access to water and food. Mice were mated aged 9 weeks or remained unmated as virgin matched controls. Upon identification of a copulatory plug, female mice were fed a standard chow diet (CRM, Special Diets Services) or chow supplemented with 0.5% cholic acid (C1129, Sigma, UK) (CA diet) and virgin controls were fed matched diets for the same experimental duration. Tissues and plasma were collected from pregnant females sacrificed on day 14 or 15 of gestation (*n* = 11/group, cohort 1; *n* = 8/group, cohort 2, respectively) or age‐matched virgin mice fed chow or 0.5% CA diet for 14 or 15 days (*n* = 9/group, cohort 1; *n* = 8/group, cohort 2). Mice were fasted for 4 h from 09:00 to 13:00 with free access to water prior to sacrifice by CO_2_ asphyxiation. All animal procedures were carried out in accordance with the Animals (Scientific procedures) Act 1986 of the UK Government.

### 2.2 Isolation of primary mouse adipocytes

Adipose tissue from cohort 1 was dissected and placed in chilled PBS on ice. The tissue was thoroughly minced with scissors on ice and transferred to chilled collagenase buffer (3 mg Collagenase Type 2 (C2‐BIOC, Sigma‐Aldrich, UK) per 1 mL of PBS (Sigma‐Aldrich, UK)). Tissues were incubated in a shaker (120 rpm) at 37°C for 1 h. Afterwards, the cell suspension was passed through a 100 μm sieve to remove undigested fragments. The cell slurry was centrifuged at 500*g* for 10 min at 4°C to separate the adipocyte and stromal vascular fraction. The adipocyte fraction was collected and lysed with 1 mL of QIAzol (79306, Qiagen, UK) following which RNA was extracted.

### 2.3 RNA isolation, cDNA synthesis, and quantitative real‐time PCR analysis

Total adipocyte RNA was extracted using RNeasy Lipid Tissue Mini Kit (74804, Qiagen, UK) and reverse transcribed into cDNA using SuperScript II Reverse Transcriptase (18064014, Thermo Fisher Scientific, UK) in accordance with the manufacturers' instructions. Real‐time quantitative PCR was performed on a Viia7 system (Thermo Fisher Scientific, UK) using SYBR Green Mastermix (A46012, Sigma‐Aldrich, UK). Relative mRNA levels were calculated using the comparative Ct method normalized to 18S housekeeper. Primer sequences are as follows: *18S* – Forward: GTA ACC CGT TGA ACC CCA TT; Reverse: CCA TCC AAT CGG TAG TAG CG. *ATGL* – Forward: GGA GAC CAA GTG GAA CAT CTC A; Reverse: AAT AAT GTT GGC ACC TGG TTC A. *CD‐36* – Forward: TGG TGG ATG GTT TCC TAG CC; Reverse: TAC GTG GCC CGG TTC TAA TTC. *CGI‐58* – Forward: GCG GTG ATG AAA GCG ATG G; Reverse: AGG TGA CTA ACC CCT CCA CC. *FABP4* – Forward: ACA CCG AGA TTT CCT TCA AAC TG; Reverse: CCA TCT AGG GTT ATG ATG CTC TTC A. *Glut4* – Forward: GCC CGG ACC CTA TAC CCT AT; Reverse: GGG TTC CCC ATC GTC AGA G. *HSL* – Forward: CTA TTC AGG GAC AGA GGC AG; Reverse: TAG TTC CAG GAA GGA GTT GAG. *LPL* – Forward: TCG TCA TCG AGA GGA TCC GA; Reverse: TGT TTG TCC AGT GTC AGC CA. *PLIN2* – Forward: GCT CTC CTG TTA GGC GTC TC; Reverse: TTG GCC ACT CTC ATC ACC AC. *SREBP2* – Forward: CAC AAT ATC ATT GAA AAG CGC TAC CGG TCC; Reverse: TTT TTC TGA TTG GCC AGC TTC AGC ACC ATG. *VLDLR* – Forward: TAG CTG TGG ATC CGT TGT CG; Reverse: CGG CTT TTG ACA AGG TCG AG.

### 2.4 Blood biochemistry

Plasma samples from cohort 1 were analyzed using Beckman Synchron LX20 chemistry analyzer (Beckman Coulter, UK) and commercially available kits were used for the measurement of total cholesterol (467825, Beckman Coulter, UK), HDL‐cholesterol (467820, Beckman Coulter, UK), and triglycerides (445850, Beckman Coulter, UK). Measurements were normalized to tissue protein content as previously described (Papacleovoulou et al., 2013). Assays were performed in the Laboratory for Health Protection Research, National Institute of Public Health and the Environment, Netherlands. Blood samples from cohort 2 were measured for blood glucose after a 4 hour fast using a Freestyle Freedom Lite monitor (Abbott Laboratories Ltd.).

### 2.5 Ultra‐performance liquid chromatography–tandem mass spectrometry

Plasma samples from cohort 1 were analyzed by ultra‐performance liquid chromatography‐tandem mass spectrometry (UPLC‐MS/MS) as previously described (Abu‐Hayyeh et al., 2016; Makki et al., 2023). Assays were performed in The Wallenberg Laboratory, Department of Molecular and Clinical Medicine, Sahlgrenska Academy, University of Gothenburg, Gothenburg, Sweden.

### 2.6 Adipokine antibody array

20 μL plasma were pooled from 6 mice per group (120 μL total) and processed as a single sample. The assay was performed according to the manufacturer's instructions (ARY013, R&D Systems). Blots were developed on X‐ray films and pixel density analyzed using ImageJ software.

### 2.7 Statistical analysis

Results were analyzed using Graphpad Prism 10.2.3. All values were expressed as mean ± SD for biological replicates. Values were analyzed for statistical significance using a two‐way ANOVA with Tukey's multiple comparisons test to determine significance between each group, or an unpaired *t*‐test with Welch's correction for comparing two groups.

## 3 RESULTS

**FIGURE 1:**
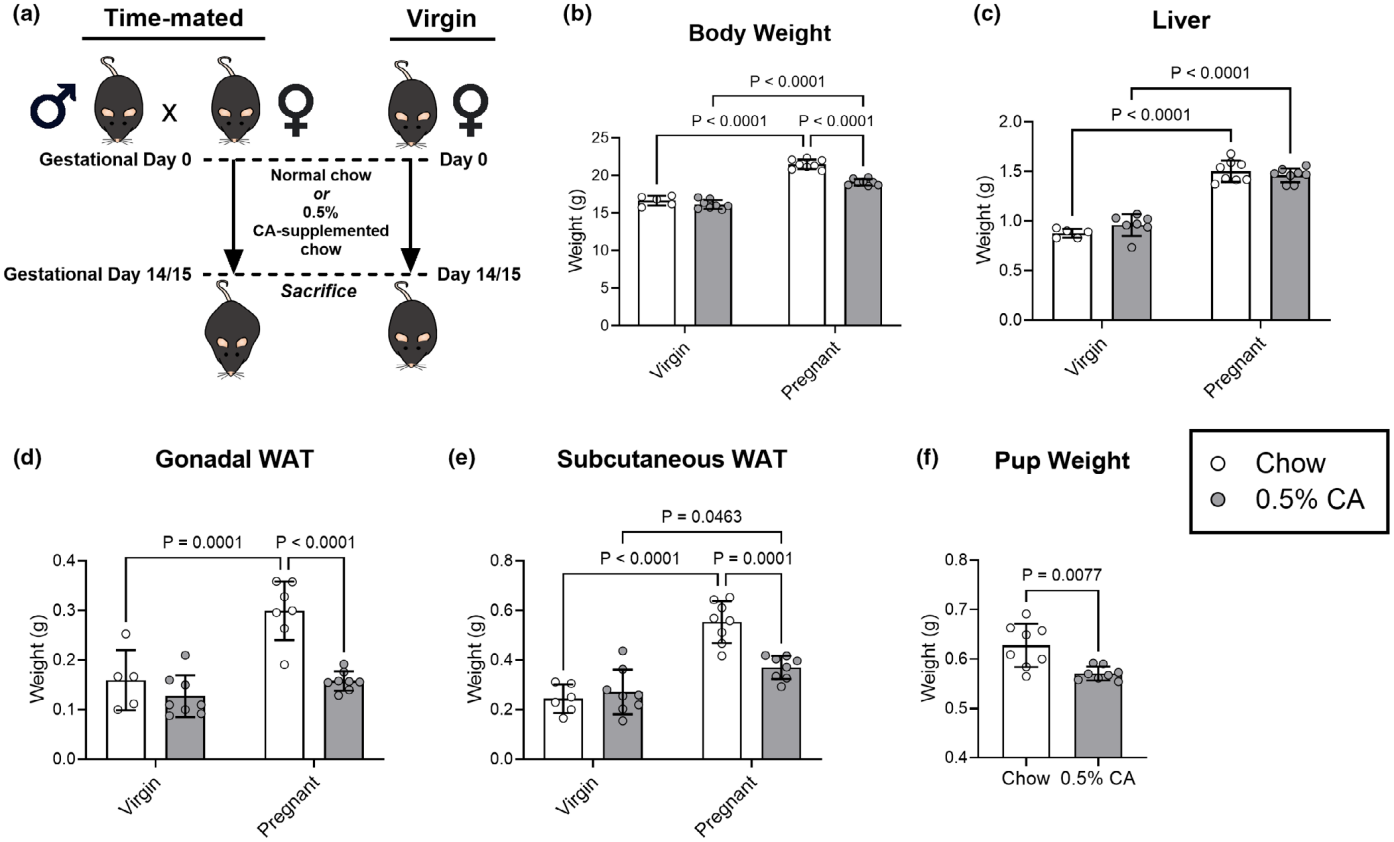
Body and organ weight differ in pregnancy with CA dietary supplementation. (a) Virgin or pregnant mice were fed standard chow or 0.5% cholic acid (CA)‐supplemented diet from identification of copulatory plug until gestational day 14 (cohort 1) or 15 (cohort 2) when the mice were sacrificed. Virgin mice were fed matched diets for equivalent numbers of days. Weights from mice at gestational day 15 or matched virgin mice were measured for: (b) whole body weight, excluding the uterine horn and fetus weight, (c) liver, (d) gonadal white adipose tissue (WAT), (e) subcutaneous WAT. (f) Estimated average pup weight per pregnancy. White bars/circles: Standard chow; gray bars/circles: 0.5% CA‐supplemented chow. Results are expressed as mean ± standard deviation. Statistical differences were assessed using a two‐way ANOVA for all maternal measurements and a Welch's *t*‐test for pup weight. Significant *p* values are indicated on each graph.

To investigate the impact of hypercholanemia on lipid metabolism and adipose tissue function during pregnancy, mice were fed a 0.5% cholic‐acid (CA) supplemented diet or standard chow from identification of a copulatory plug up to sacrifice on gestational day 14 (cohort 1) or 15 (cohort 2). Virgin females were fed the same CA diet or standard chow for a matched number of days (Figure 1a). Total body weight, excluding the fetuses and uterine horn, was increased in pregnant mice, with CA supplementation reducing this weight gain, suggesting that the excess bile acids interfered with pregnancy‐specific weight gain but did not interfere with body weight in nonpregnant mice (Figure 1b). Liver weight increased during pregnancy and was unaltered by CA feeding (Figure 1c). Gonadal and subcutaneous white adipose tissue (WAT) depots were both unaltered in virgin mice with CA diet, although the adipose depots were decreased in pregnant CA‐fed mice by 47% and 33%, respectively, compared to their chow‐fed counterparts (Figure 1d,e). The average pup weight per pregnancy, estimated from uterine horn weight divided by the number of pups per litter, was reduced by approximately 10% in dams fed CA (Figure 1f), without a decrease in litter number (mean ± standard deviation: 7.4 ± 1.3 pups in chow pregnancies vs. 6.6 ± 0.5 pups in CA‐supplemented pregnancies, *p* = 0.164). Since our previous work did not demonstrate a reduction in food intake (Milona et al., 2010) and only adipose weight gain during pregnancy was altered in this experiment, we investigated how hypercholanemia might alter pregnancy‐associated adipose tissue expansion and lipid metabolism.

**FIGURE 2:**
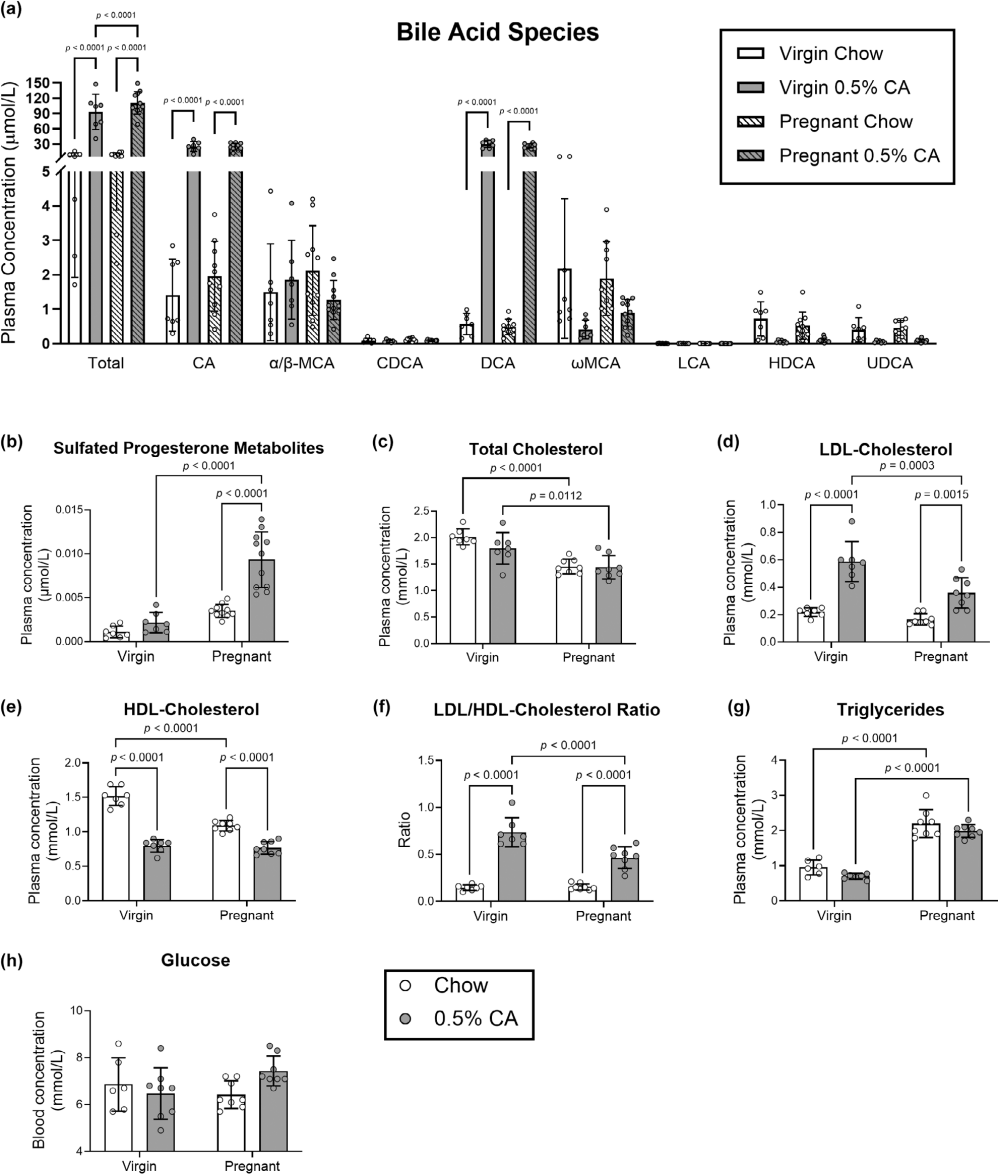
Plasma profile is altered by pregnancy and cholic acid supplementation. Mated mice were fed standard chow diet or diet supplemented with 0.5% cholic acid from identification of a copulatory plug until gestational day 14. Virgin mice were fed matched diets for 14 days (cohort 1). Mice were samples after a 4 h fast and analyzed for: (a) individual bile acid species (virgin chow: White bars/circles; virgin 0.5% CA: Gray bars/circles; pregnant chow: Striped white bars; pregnant 0.5% CA: Striped gray bars), (b) progesterone sulfate metabolites, (c) total cholesterol, (d) LDL‐cholesterol, (e) HDL‐cholesterol, (f) LDL/HDL‐cholesterol ratio, and (g) triglycerides. (h) Blood glucose was analyzed from mice sacrificed on day 15 of gestation and cholic acid feeding (or matched virgin/chow‐fed mice) after a 4‐h fast (chow: White bars; 0.5% CA: Gray bars). Results are expressed as mean ± standard deviation. Statistical analysis was performed by a two‐way ANOVA with uncorrected Fisher's least significant difference test. Significant *p* values are indicated on each graph. CA, cholic acid; CDCA, chenodeoxycholic acid; DCA, deoxycholic acid; HDCA, hyodeoxycholic acid; HDL high‐density lipoprotein; LCA, lithocholic acid; LDL, low‐density lipoprotein; MCA, muricholic acid; UDCA, ursodeoxycholic acid.

We confirmed that CA supplementation in the diet altered the plasma bile acid composition through UPLC‐MS/MS in cohort 1. Total plasma bile acids were increased in mice fed 0.5% CA diet in both virgin and pregnant mice to a similar degree, with no pregnancy‐related effect observed (Figure 2a). Addition of CA in the diet increased primary bile acid CA and its microbially‐metabolized derivative secondary bile acid deoxycholic acid (DCA). A reduction was observed in low‐abundance secondary bile acid species ω‐muricholic acid (ωMCA), hyodeoxycholic acid (HDCA), and ursodeoxycholic acid (UDCA), whereas plasma concentrations of primary bile acids α/β‐MCA and chenodeoxycholic acid (CDCA) were unaffected. Sulfated progesterone metabolites are increased in human pregnancy and further in pregnancies complicated by ICP (Abu‐Hayyeh et al., 2016). Levels of sulfated progesterone metabolites were elevated both by pregnancy and CA supplementation, with an additive effect of both pregnancy and diet (Figure 2b), although the plasma concentration is over two orders of magnitude less than in serum of pregnant women.

After confirming CA supplementation caused increased plasma bile acids, we investigated how this impacted the lipid profile. Pregnancy reduced total plasma cholesterol, but CA supplementation did not alter the total plasma cholesterol in either virgin or pregnant mice (Figure 2c). LDL‐cholesterol concentrations were significantly increased (Figure 2d), and HDL‐cholesterol decreased (Figure 2e) following CA feeding irrespective of pregnancy status, which led to an increased LDL/HDL‐cholesterol ratio (Figure 2f). Plasma triglyceride levels were increased with pregnancy but were unaltered by CA diet (Figure 2g). Fasting blood glucose was unaltered by CA feeding in virgin mice or by pregnancy (Figure 2h).

**FIGURE 3:**
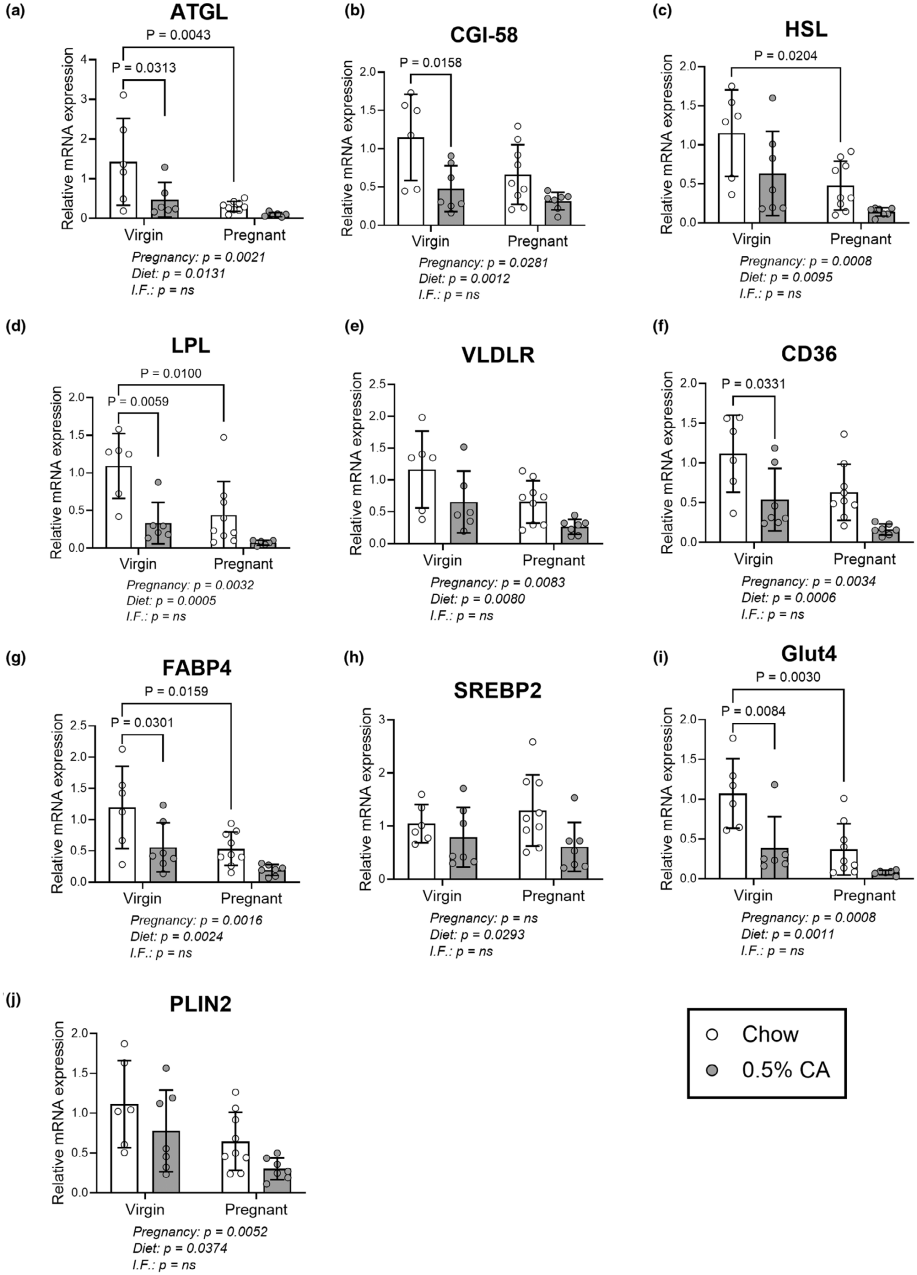
mRNA levels in gonadal adipocytes are reduced by pregnancy and cholic acid feeding. Mated mice were fed standard chow diet (white bars) or diet supplemented with 0.5% cholic acid (CA, gray bars) from identification of a copulatory plug until gestational day 14. Virgin mice were fed matched diets for 14 days (cohort 1). Mature adipocytes extracted from gonadal white adipose tissue were analyzed for mRNA levels of: (a) ATGL, (b) CGI‐58, (c) HSL, (d) LPL, (e) VLDLR, (f) CD36, (g) FABP4, (h) SREBP2, (i) Glut4, and (j) PLIN2. Results are expressed as mean ± standard deviation. Statistically significant differences were tested using a two‐way ANOVA with Tukey's multiple comparison test. *p* values for the effect of pregnancy, diet and interaction factor (I.F.) between pregnancy and diet are shown below each graph. Significant *p* values are indicated on each graph. ATGL, adipose triglyceride lipase; CD36, cluster of differentiation 36; CGI‐58, coactivator comparative gene identification‐58; FABP4, fatty acid binding protein 4; Glut4, Glucose transporter 4; HSL, hormone serine lipase; I.F., interaction factor, LPL, lipoprotein lipase; PLIN2, perilipin 2; SREBP2, sterol regulatory element binding transcription factor 2; VLDLR, very low‐density protein lipase receptor.

**FIGURE 4:**
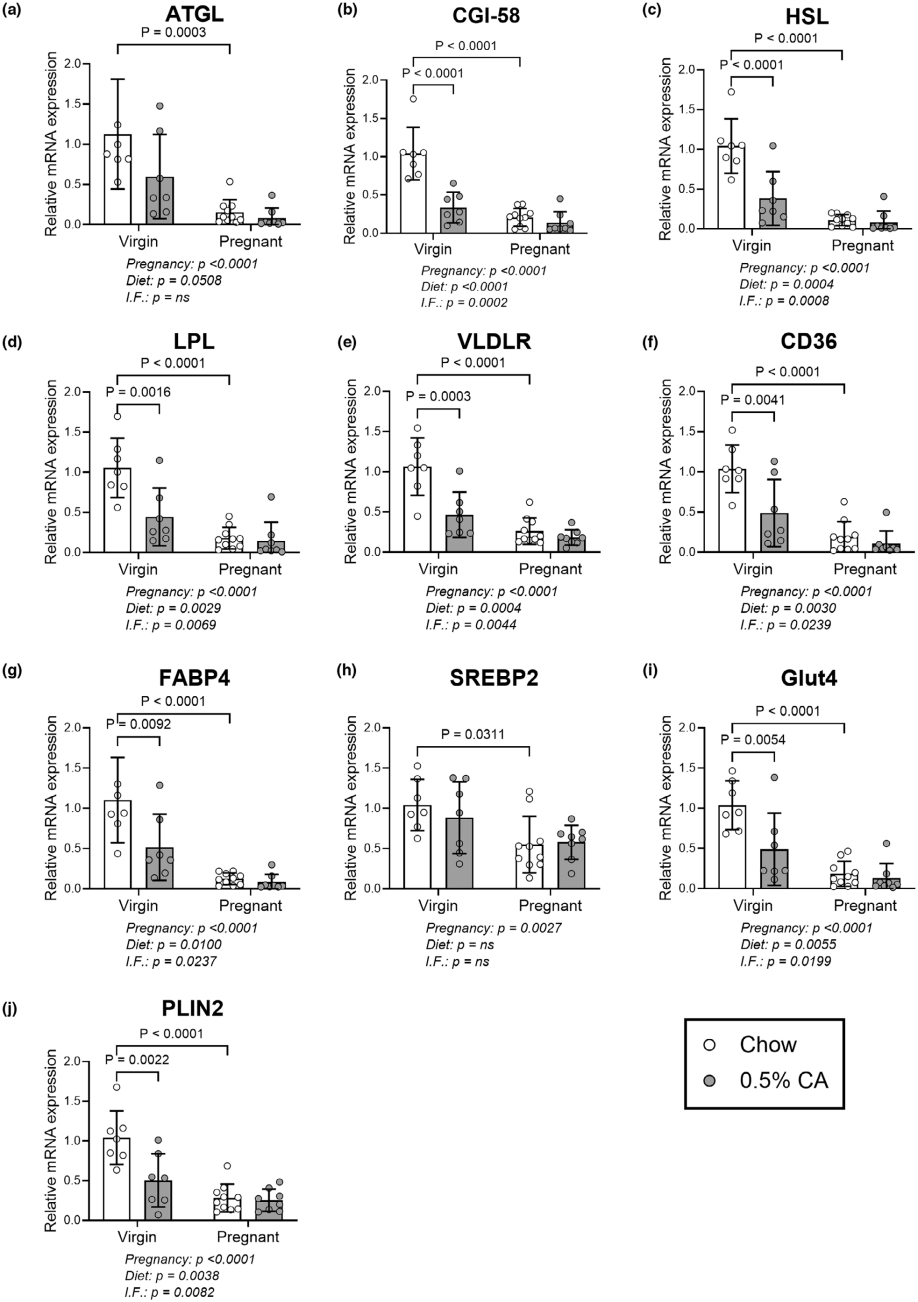
mRNA levels in subcutaneous adipocytes are reduced by pregnancy and cholic acid feeding. Mated mice were fed standard chow diet (white bars) or diet supplemented with 0.5% cholic acid (CA, gray bars) from identification of a copulatory plug until gestational day 14. Virgin mice were fed matched diets for 14 days (cohort 1). Mature adipocytes extracted from subcutaneous white adipose tissue were analyzed for mRNA levels of: (a) ATGL, (b) CGI‐58, (c) HSL, (d) LPL, (e) VLDLR, (f) CD36, (g) FABP4, (h) SREBP2, (i) Glut4, and (j) PLIN2. Results are expressed as mean ± standard deviation. Statistically significant differences were tested using a two‐way ANOVA with Tukey's multiple comparison test. *p* values for the effect of pregnancy, diet and interaction factor (I.F.) between pregnancy and diet are shown below each graph. Significant *p* values are indicated on each graph. ATGL, adipose triglyceride lipase; CD36, cluster of differentiation 36; CGI‐58, coactivator comparative gene identification‐58; FABP4, fatty acid binding protein 4; Glut4, Glucose transporter 4; HSL, hormone serine lipase; LPL, lipoprotein lipase; PLIN2, perilipin 2; SREBP2, Sterol regulatory element binding transcription factor 2; VLDLR, very low‐density protein lipase receptor.

Since the levels of HDL‐ and LDL‐cholesterol were altered by CA diet, we explored the impact on the mRNA levels of genes involved in adipocyte lipid metabolism and transport. In gonadal adipocytes, all transcript levels showed a significant influence of both pregnancy and diet (Figure 3) apart from sterol regulatory binding transcription factor 2 (SREBP2) (Figure 3h), which only showed an effect from CA diet administration. Transcript levels for adipose triglyceride lipase (ATGL) which cleaves the first fatty acid (FA) from triglycerides from lipid stores, together with coactivator comparative gene identification‐58 (CGI‐58), were suppressed by CA feeding in virgin mice (Figure 3a,b) alongside a reduction in pregnancy. mRNA for hormone serine lipase (HSL), which cleaves the second FA from diglycerides, was reduced in pregnancy (Figure 3c). Transcript levels of lipoprotein lipase (LPL) were reduced in nonpregnant mice fed CA diet, and in pregnant mice fed control diet (Figure 3d), whereas there were no significant differences between the groups in very low‐density lipoprotein receptor (VLDLR) expression (Figure 3e). Cluster of differentiation 36 (CD36), which imports long‐chain FAs from the blood into the cell, showed significant reduction in mRNA expression with CA diet administration (Figure 4f). Transcript levels for fatty acid binding protein 4 (FABP4), a chaperone for FAs within the cell, and glucose transporter 4 (Glut4), which imports glucose from the blood stream, were reduced by both CA feeding in the nonpregnant mouse, and by pregnancy (Figure 3g,i). mRNA expression of SREBP2, a master regulator of sterol and lipid synthesis, alongside perilipin 2 (PLIN2) were not significantly affected in any condition (Figure 3h,j). No interaction between pregnancy and diet was observed for any transcripts investigated although a trend for further reduction in CA‐fed pregnant mice was apparent.

mRNA expression in subcutaneous adipocytes showed a similar trend to those of gonadal adipocytes. Both pregnancy and CA diet affected mRNA expression in all transcripts investigated, alongside an interaction between pregnancy and diet (Figure 4a–g,i,j), excepting SREBP2 which only showed an effect of pregnancy (Figure 4h). Pregnancy in mice fed a standard diet showed a significant reduction in subcutaneous adipocyte mRNA levels for all targets by 83%–90%. CA diet in nonpregnant mice also demonstrated a 47%–68% decrease in abundance in all transcripts except for ATGL. Whilst there were no significant reductions in pregnant mice fed CA, all relative expression values trended lower than for their pregnant chow‐fed counterparts.

Thus, the mRNA levels of many genes involved in lipid homeostasis in both subcutaneous and gonadal adipocytes were reduced by both pregnancy and CA administration. The effect of CA diet was comparable to that of late pregnancy in gonadal adipocytes, however, pregnancy had a greater suppressive effect in subcutaneous adipocytes.

**FIGURE 5:**
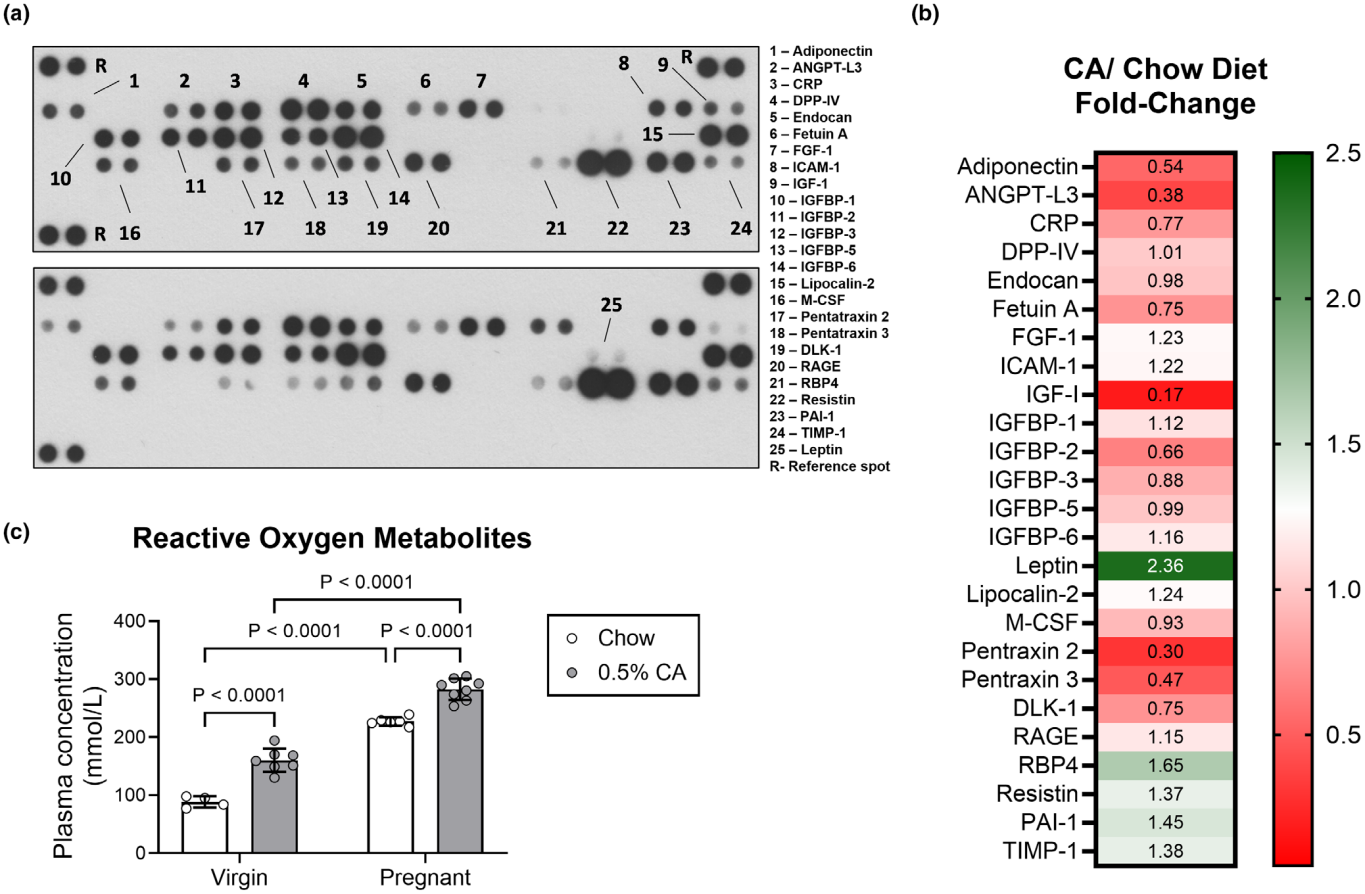
Cholic acid supplementation in pregnancy alters the plasma adipokine profile and reactive oxygen metabolites. Plasma from pregnant mice fed standard chow or chow supplemented with 0.5% cholic acid (CA) at gestational day 14 were assessed for adipokine expression and reactive oxygen metabolites. (a) Adipokine array in plasma. Samples from 6 mice were pooled and run on a single array. Blots for CA‐fed and chow‐fed mice were taken on the same exposure. Pairs of targets (1‐25) and reference spots (R) are labeled in the key. (b) Mean fold‐change of expression of adipokine array targets in plasma. All fold‐changes were analyzed from the exposure shown in a. except for Leptin (25) which was analyzed from a longer exposure (Figure S1). (c) Levels of plasma reactive oxygen metabolites in virgin and pregnant mice fed either normal chow (white bars) or 0.5% CA diet (gray bars) for 14 days. Results are expressed as mean ± standard deviation. ANGTP‐L3, angiopoietin‐like protein‐3; CRP, C‐reactive protein; DLK‐1, delta like noncanonical Notch ligand 1; DPP‐IV, dipeptidyl peptidase‐IV; FGF‐1, fibroblast growth factor‐1; ICAM‐1, intracellular adhesion molecule 1; IGF‐1, insulin‐like growth factor 1; IGFBP, insulin‐like growth factor binding protein; M‐CSF, macrophage colony‐stimulating factor; PAI‐I, plasminogen activator inhibitor‐1; R, reference spot; RAGE, receptor for advanced glycation endproducts; RBP‐4, retinol‐binding protein 4; TIMP‐1, tissue inhibitor of metalloproteinase‐1. Statistical significance was assessed by a two‐way ANOVA with uncorrected Fisher's LSD post hoc analysis. Significant *p* values are indicated on each graph.

To investigate changes in adipose signaling, we performed an adipokine array using pooled plasma samples from pregnant mice. Mice fed CA diet showed a 2.36‐fold induction in leptin, involved in energy homeostasis, metabolism, and immune function (Park & Ahima, 2015) (Figure 5a,b). CA supplementation also induced a 1.45‐fold increase in plasminogen activator inhibitor‐1 (PAI‐1) which regulates the fibrinolytic system and whose expression is induced by pro‐inflammatory cytokines (Morrow et al., 2021), and a 1.65‐fold increase in retinol binding protein 4 (RBP4) that is known to be increased in mouse models of obesity and insulin resistance (Tamori et al., 2006). Circulating pentraxin‐2 and ‐3, factors that modulate the innate immune system (Clos, 2013), were reduced by over 50%. CA administration during pregnancy caused a reduction of insulin‐like growth factor 1 (IGF‐1) to 0.17‐fold that of mice on chow diet. IGF‐1 is also known to regulate lipid metabolism and inflammation‐modulating hormone adiponectin (Khoramipour et al., 2021) which was reduced to almost half in CA‐fed mice. Circulating angiopoietin‐like protein 3 (ANGTP‐L3), which inhibits lipoprotein lipase and whose deficiency reduces plasma cholesterol and triglycerides (Tarugi et al., 2019), was reduced by over 60% in pregnant mice fed CA. CA supplementation in virgin mice doubled plasma reactive oxygen metabolites (ROM). Pregnancy itself caused an increase in ROM beyond this, with CA addition during pregnancy exacerbating ROM levels further (Figure 5c). Together, these data point to a dysregulation in adipokine homeostasis promoting an increase in circulating inflammatory markers and oxidative stress and suggest that bile acid overload induces oxidative and inflammatory imbalance during pregnancy.

## 4 DISCUSSION

The increase in fat mass during pregnancy is critical in preparation for late gestational metabolic changes where maternal tissues increase lipid oxidation to maintain a high glucose supply to the fetuses and to ensure sufficient lipids are available postpartum for lactation. We observed that bile acid overload impaired white adipose tissue expansion during pregnancy and suppressed most adipocyte transcripts in pregnant mice additive to the pregnancy‐related reduction in transcript levels observed. We did not measure food intake in these mice, and therefore cannot rule out that changes observed may be due to reduced dietary intake. One explanation for the reduced adipose tissue weight could be that bile acids are interfering with nutrient uptake, although other studies have suggested CA supplementation increases cholesterol absorption in humans (Woollett et al., 2004) and increases hepatic cholesterol accumulation in mice (Murphy et al., 2005). Against this explanation, however, is that adipose tissue but not liver failed to expand in CA‐fed pregnant mice, and no change in adipose weight was observed in non‐pregnant mice fed CA diet. Furthermore, the increase in plasma LDL‐cholesterol and unaltered plasma glucose in pregnant CA‐fed mice are not consistent with these mice having low nutrient intake. Therefore, we suspect that bile acids specifically interfere with adipose expansion. Bile acids are also known to promote thermogenesis in brown adipose tissue (Zietak & Kozak, 2015) and to beige (convert to a brown adipocyte‐like state) white adipocytes (Velazquez‐Villegas et al., 2018) which could cause an increase in energy expenditure to account for the reduced pregnancy‐associated weight gain.

A reduction in transcripts of both lipogenic and lipolytic genes was previously noted in the white and brown adipose tissue of *Fxr* ^*−/‐*^ *Shp* ^*−/−*^ male mice (Zhou et al., 2023), consistent with our data. The pregnancy‐related reduction in adipocyte mRNAs might be due to metabolic changes occurring in late pregnancy, although further work is required to investigate whether differences in adipose transcript levels are observed in early pregnancy and how CA supplementation may alter adipose expansion and transcript expression at this earlier stage. Future studies would also benefit from histological understanding of the impact of hypercholanemia on adipocyte expansion over the course of gestation alongside the subcellular localization and abundance of certain proteins such as Glut4 or lipid import proteins. In late gestation, the effect of pregnancy on adipose tissue transcript suppression is much larger than the effect of CA diet, which was only observed in some transcripts. We hypothesize that during early gestation CA administration interferes with adipose depot lipogenesis and lipid import, but that the observed suppression of adipose depot genes in late gestation seen here in both chow‐ and CA‐fed mice is reflective of the alteration of maternal metabolism to provide sufficient lipids for fetal consumption. Currently, published work is lacking on normal pregnancy‐related changes in adipose depots in murine models, and future work should focus on adipose depot changes during gestation to understand how this tissue is altered during healthy and complicated pregnancies.

The increase in circulating LDL‐cholesterol in CA‐fed pregnant mice could indicate increased availability for placental transfer (Figure 2d,h) (Borges Manna et al, 2020; Murphy et al., 2005; Woollett et al., 2004). CA administration increases LDL‐cholesterol and reduces HDL‐cholesterol regardless of pregnancy state (Figure 2c,d) but did not significantly alter fasting glucose in pregnant mice (Figure 2h). Currently, it is unknown whether the dyslipidemia observed is secondary to changes in insulin signaling and examining changes over the course of pregnancy would address these questions. We hypothesize that the reduction in bile flow and reduced de novo synthesis of bile acids from cholesterol that occurs in cholestatic mice (Li & Apte, 2015) leads to buildup of excessive hepatic cholesterol which results in an increase in export via (V)LDL‐cholesterol alongside a reduction in import which may explain the reduced HDL‐cholesterol concentrations. Our previous work administering a 0.5% CA diet to dams from identification of copulatory plug until gestational day 18 showed alterations in the fetal lipid profile. Total fetal serum cholesterol and LDL‐cholesterol was significantly increased in the study by Pataia et al. (2020) although unchanged in the study by Borges Manna et al. (2020). Reduced fetal HDL‐cholesterol and increased triglycerides were observed in both studies, suggesting that fetal liver health may be impacted by gestational hypercholanemia and/or the dyslipidemia that accompanies it. In our model, hypercholanemia and dyslipidemia did not lead to increased fetal weight as has been observed in a model of hypercholesterolemia alone (Dumolt et al., 2019). This suggests that increased circulating maternal LDL‐cholesterol is not sufficient to increase fetal weight gain in combination with elevated circulating bile acids. Hypercholanemia has been suggested to interfere with placental function (Geenes et al., 2011; Lin et al., 2023; Perez et al., 2006) and could account for the lower fetal weight in CA‐fed dams.

A recent publication which administered CA orally from mid to late gestation in mice also observed fetal growth restriction, which was suggested to be downstream of reactive oxygen species‐mediated activation of the placental general control nonderepressible 2/eukaryotic initiator factor α pathway (Lin et al., 2023). We observed an increase in plasma ROM alongside an increase in immune‐modulating pentraxins, and an increase in PAI‐1, which is upregulated by pro‐inflammatory cytokines (Figure 5). Together, these suggest that CA administration in pregnancy results in a shift towards a pro‐inflammatory and pro‐oxidative environment. It may be that the inflammatory environment resulting from bile acid overload, rather that the bile acids directly, are responsible for the fetal growth restriction. Alternatively, maternal IGF‐1 has been observed to correlate with birth weight and placental weight (Luo et al., 2012), and the 5‐fold reduction in IGF‐1 in pregnant CA‐fed mice may play a role in this phenomenon. The placenta can indirectly influence maternal IGF‐1 through secretion of placental growth hormone (Alsat et al., 1998) and therefore placental changes could reduce maternal IGF‐1 production. Alternatively, if the CA‐fed mice had reduced food intake, this could impact placental growth hormone and its subsequent impact to maternal IGF‐1 (Newbern & Freemark, 2011). Further investigation into the primary cause of maternal IGF‐1 reduction and its potential role in cholestatic pregnancy is warranted. The fetal growth restriction observed in this and another model (Lin et al., 2023) may not be translatable to human pregnancies. Infant birth weight, when adjusted for gestational age at birth, has been reported as unaltered (Arthuis et al., 2020) or with an increased risk for large for gestational age babies (Chen et al., 2024; Martineau et al., 2014; Wikström Shemer et al., 2013). One explanation for the differences observed could be the multifetal nature of murine pregnancies and different nutrient demands.

Leptin canonically correlates with adipose mass (Park & Ahima, 2015) but this does not appear to be the case in our mouse model of hypercholanemic pregnancy as we observed a reduction in adipose tissue weight concomitant with a 2.36‐fold increase in plasma leptin levels. Elevated leptin has been observed in other animal models of cholestasis and appears to exacerbate the disease (DeMorrow et al., 2016; Petrescu et al., 2022). Contrary to the observation in CA‐fed pregnant mice, a recent publication suggested that circulating leptin is reduced in women with ICP, particularly those with higher bile acids, whereas adiponectin concentrations are elevated (Yurtcu et al., 2022). This could reflect the hypercholesterolemia and hypertriglyceridemia (Dann et al., 2006; Martineau et al., 2014) observed in women with ICP compared to the milder effects observed in murine plasma cholesterol and unaltered triglycerides, although adipose tissue function in ICP remains to be explored.

This work highlights critical dysfunction in adipose tissue weight gain and gene expression during hypercholanemic pregnancy in mice, combined with an increased inflammatory landscape and aberrant adipokine homeostasis that may contribute to the observed fetal growth restriction. Further work is needed to unravel the interplay between bile acid overload, adipose tissue dysfunction, and inflammation. Future experiments should investigate whether the altered maternal adipose tissue landscape impacts the fetal lipidome, and whether this phenomenon is mirrored in women with ICP and their babies.

## AUTHOR CONTRIBUTIONS

VN, GP, and CW contributed to conception and design of the research. VN and EB performed the animal experiments. VN performed the adipocyte extraction and adipokine assay, ALM performed the qPCR. EJ performed the plasma lipid and ROM analysis. POB and HUM performed the mass spectrometry analysis. ALM and VN analyzed the data and ALM drafted the manuscript. All authors contributed to the editing of the manuscript except for HUM who passed away prior to manuscript production. CW is the guarantor of this work and has had full access to all study data to ensure data interpretation and accuracy.

## FUNDING INFORMATION

This research was funded in by the Wellcome Trust (Programme Grant 092993/Z/10/Z) and an NIHR Senior Investigator awarded to CW (Reference NIHR200254). The views expressed are those of the authors and not necessarily those of the funders.

## CONFLICT OF INTEREST STATEMENT

VN, ALM, EB, EJ, GP, and POB declare no conflicts of interest. HUM declares consulting or advisory board activities for Albireo, Calliditas, Intercept, Mirum and Zealand and lecture fees by Albireo, Intercept and Bayer. CW is a consultant for Mirum Pharmaceuticals and GSK.

## ETHICS STATEMENT

All animal procedures were carried out in accordance with the Animals (Scientific procedures) Act 1986 of the UK Government. Approval was granted for these experiments by the Home Office.

## Supporting information


Figure S1.

## Data Availability

All original data are available upon reasonable request.
