# Supplementary material for: Gestational hypercholanemia suppresses pregnancy‐associated adipose mass increase and stimulates a pro‐inflammatory environment in mice
Source: Physiol Rep. 2024 Dec 12;12(23):e70141. doi: 10.14814/phy2.70141 (PMC11637612; doi:10.14814/phy2.70141)
Supplement: Supplementary file 1 — Figure S1. [file PHY2-12-e70141-s001.docx]

**Supplementary Figures**


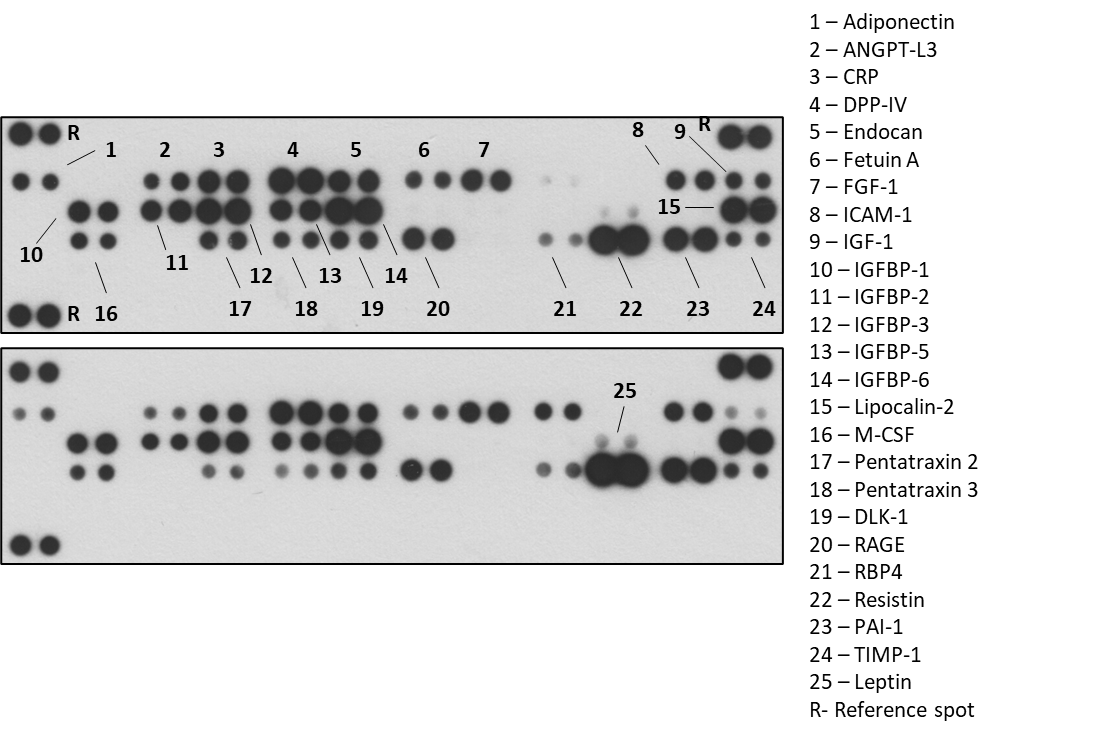


**Supplementary Figure 1:** Longer exposure of adipokine array in plasma samples from pregnant mice fed either standard chow or chow supplemented with 0.5% cholic acid (CA) from identification of copulatory plug until sacrifice at gestational day 14. Adipokines were assayed in duplicate from pooled plasma samples. Each detectable adipokine is marked numerically on the blot with the corresponding adipokine labelled in the key to the right. ANGTP-L3, angiopoietin-like protein-3 ; CRP, C-reactive protein; DLK-1, delta like non-canonical Notch ligand 1; DPP-IV, dipeptidyl peptidase-IV; FGF-1, fibroblast growth factor-1; ICAM-1, intracellular adhesion molecule 1; IGF-1, insulin-like growth factor 1; IGFBP, insulin-like growth factor binding protein; M-CSF, macrophage colony-stimulating factor; PAI-I, plasminogen activator inhibitor-1; R, reference spot; RAGE, receptor for advanced glycation endproducts; RBP-4, retinol-binding protein 4; TIMP-1, tissue inhibitor of metalloproteinase-1.
